# Metamorphic proteins: the Janus proteins of structural biology

**DOI:** 10.1098/rsob.210012

**Published:** 2021-04-21

**Authors:** Kulkarni Madhurima, Bodhisatwa Nandi, Ashok Sekhar

**Affiliations:** Molecular Biophysics Unit, Indian Institute of Science, Bangalore 560 012, India

**Keywords:** metamorphic proteins, NMR spectroscopy, protein evolution, protein dynamics, structure–function relationship, fold-switching

## Abstract

The structural paradigm that the sequence of a protein encodes for a unique three-dimensional native fold does not acknowledge the intrinsic plasticity encapsulated in conformational free energy landscapes. Metamorphic proteins are a recently discovered class of biomolecules that illustrate this plasticity by folding into at least two distinct native state structures of comparable stability in the absence of ligands or cofactors to facilitate fold-switching. The expanding list of metamorphic proteins clearly shows that these proteins are not mere aberrations in protein evolution, but may have actually been a consequence of distinctive patterns in selection pressure such as those found in virus–host co-evolution. In this review, we describe the structure–function relationships observed in well-studied metamorphic protein systems, with specific focus on how functional residues are sequestered or exposed in the two folds of the protein. We also discuss the implications of metamorphosis for protein evolution and the efforts that are underway to predict metamorphic systems from sequence properties alone.

## Graphical abstract

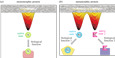

## 1. Introduction

The single-sequence-structure–function hypothesis is the linchpin that has kept the wheels of structural biology spinning for decades after the first crystal structure of myoglobin was solved by John Kendrew and colleagues in 1958 [1]. This hypothesis states that the amino acid sequence of a protein codes for a unique native state structure, which then performs a distinct function. In the past two decades, there have been a number of discoveries that have challenged this hypothesis, to the point of rendering it obsolete. For example, we now know of the existence of intrinsically disordered proteins [2–5], which are polypeptide chains without stable secondary or tertiary structure, but that nevertheless perform vital functions through a variety of mechanisms such as folding upon binding [6] or acquiring structure following post-translational modifications [7]. We have stumbled upon moonlighting proteins [8,9] that are capable of performing more than one function using the same polypeptide sequence. We also now understand that biomolecular function is dictated not only by structure, as originally hypothesized, but also by biomolecular dynamics occurring on multiple timescales [10,11]. Finally, we have seen the emergence of metamorphic proteins, which are capable of folding into more than one unique structural topology [12]. In ancient Roman mythology, Janus was the god of transitions and duality, beginning and ending, war and peace, and arrival and departure. His dual nature was embodied in his portrayal as a god with two heads facing in opposite directions. Given their ability to adopt two or more distinct native state structures, metamorphic proteins are the Janus proteins of structural biology.

**Figure 1.**
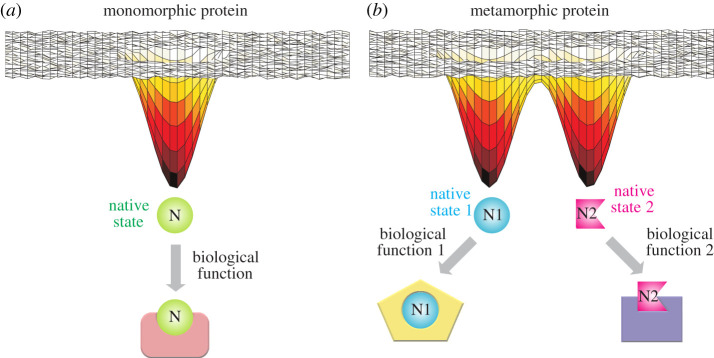
Representative conformational free energy landscapes of a protein that folds into a single structure (monomorphic, *a*) and a metamorphic protein that folds into two distinct structures (*b*). The monomorphic protein in this case has a single biological function, while the metamorphic protein can use its conformational heterogeneity to perform more than one biological function.

Metamorphic proteins exist in two or more well-defined structures in the absence of ligands or cofactors (figure 1) [13–15]. This distinguishes them from proteins that undergo conformational rearrangement subsequent to binding events. While structural heterogeneity is a hallmark of metamorphic proteins, we do not consider a protein to be metamorphic simply because it adopts a heterogeneous ensemble. For example, intrinsically disordered proteins, which rapidly interconvert between a number of similar conformations, do not qualify as metamorphic proteins because they lack stable three-dimensional structures. Within the framework of this definition, however, there is considerable variability in the features of the currently known metamorphic proteins. For example, in the case of lymphotactin [16] and IscU [17], the two structures can reversibly interconvert within approximately 1 s and also have distinct binding partners within the cell. In other cases such as KaiB [18] and Mad2 [19], it takes hours for the two conformations to interconvert, and only one of the structures has a downstream binding component. Metamorphic proteins illustrate the malleability inherent in protein-free energy landscapes and are textbook examples for why we must look beyond single static biomolecular structures if we are to understand function and malfunction in their entirety.

In this review, we first describe the key features of five metamorphic protein systems that have been structurally characterized so far: lymphotactin, KaiB, IscU, Mad2 and RfaH. We focus on the structures of the two conformations and how a single sequence is able to accommodate two native states. We also outline how the structure is able to inform the function. This set of metamorphic proteins is not exhaustive, and other proteins that have been described as metamorphic include selecase [20], MinE [21], CLIC [22] and HIV-1 reverse transcriptase [23]. Following this description of five metamorphic proteins, we detail the exciting connection between metamorphic proteins and protein evolution, as well as the efforts that have been taken to predict metamorphic proteins using sequence information alone. Finally, we point out the synergistic combination of methods that have fostered our current understanding of metamorphic proteins.

## 2. Fold-switching and its consequences in important metamorphic protein systems

### 2.1. Lymphotactin

**Figure 2.**
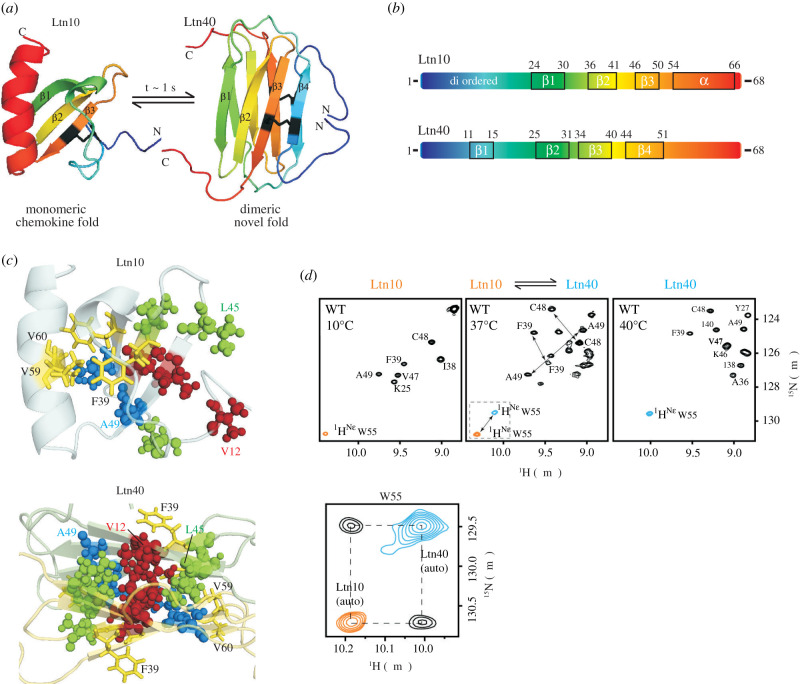
(*a*) Lymphotactin (Ltn) exists in equilibrium between an α + β chemokine fold (Ltn10, PDB ID: 1J8I) and a novel all-β dimeric fold (Ltn40, PDB ID: 2JP1). (*b*) The secondary structural elements of the Ltn10 and Ltn40 folds shown as a function of the sequence. (*c*) The Ltn40 dimer interface (bottom) is stabilized by three rows of hydrophobic residues (shown in blue, red and green spheres) running perpendicular to the monomer plane. These residues are mostly solvent-exposed in Ltn10 (top), while the residues in the core of Ltn10 (e.g. Phe39, Val59 and Val60, shown as yellow sticks) are solvent-exposed in Ltn40. (*d*) (Top) ^1^H–^15^N heteronuclear single quantum correlation (HSQC) spectra of Ltn at 10°C/200 mM NaCl (left), 37°C/150 mM NaCl (middle) and 40°C/no salt (right), where Ltn exists as Ltn10, a mixture of Ltn10 and Ltn40, and Ltn40, respectively. Phe39 and Cys48 and Ala49 backbone resonances arising from both Ltn10 and Ltn40 can be seen in the middle spectrum confirming the co-existence of the two conformations at this temperature. (Bottom) ^15^N magnetization exchange spectrum of Ltn at 37°C/150 mM NaCl, showing cross-peaks (black) linking the Ltn10 (orange) and Ltn40 (cyan) Trp55 sidechain NH resonances. The presence of these cross-peaks demonstrates that Ltn10 and Ltn40 interconvert on the ms–s timescale. Panel (*d*) is modified with permission from Tuinstra *et al*. [16].

One of the first proteins recognized to be metamorphic was the 93-residue human lymphotactin [16] (figure 2) (Ltn, also known as XCL1). Ltn is a member of the XC family of chemokines and is unusual in having only one N-terminal Cys residue and only one intramolecular disulfide bond, in contrast to members of the CC, CXC and CX3C families, which have four highly conserved Cys and two disulfide bonds each [24,25].

The fold-switching of Ltn is accompanied by a dramatic conformational rearrangement from the canonical α + β chemokine fold (Ltn10) [26], where Ltn exists as a monomer, to the novel dimeric all-β fold (Ltn40) (figure 2*a*,*b*) [16,27]. The structure of Ltn10 consists of three β-strands, Ile24–Thr30, Ala36–Thr41 and Lys46–Asp50, as well as a C-terminal α-helix from Thr54–Lys66 and a 3_10_ helix (Val21–Arg23), whereas the first 23 N-terminal residues and the C-terminal tail (Ser67--Gly93) are disordered. During the transformation to Ltn40, the α-helix unfolds, while a new β-strand forms at the N-terminus between residues 11–14, resulting in a 4-stranded Greek key fold (figure 2*a*,*b*). Virtually all inter-strand hydrogen bonds between β2–β3 and β3–β4 reorganize to accommodate the new β1, causing a shift in the registry of the β2–β3 and β3–β4 strands [16]. The dimer interface of Ltn40 is stabilized by three imposing layers of hydrophobic residues (Val12, Ile38, Val47), (Leu14, Ile40, Leu45) and (Ile29, Ala36, Ala49) arranged orthogonal to the monomer plane, most of which are solvent-exposed in Ltn10 (figure 2*c*). In contrast, the hydrophobic core of Ltn10 comprises residues Tyr27, Phe39, Val59 and Val60 that end up being solvent-exposed in Ltn40. This inversion in the degree of solvent exposure of Ltn residues gives the impression that the protein has been turned inside-out during fold-switching.

The chemokine fold is populated at 10°C and 200 mM NaCl, while the all-β structure is preferred at 40°C in the absence of salt (figure 2*d*). Intriguingly, under physiological conditions (37°C and 150 mM NaCl), Ltn10 and Ltn40 are equally populated, and interconvert rapidly and reversibly between each other with a rate constant of approximately 1 s^−1^. The increase in ionic strength is believed to favour the conversion of Ltn40 to Ltn10 by rupturing a key salt bridge between Lys25 and Glu31 that stabilizes the dimeric state. The exchange between Ltn10 and Ltn40 is visible in ^15^N magnetization transfer experiments (figure 2*d*, bottom) [16] and confirms that the two forms are not consequences of static heterogeneity in the sample.

Ltn is a highly unlikely candidate for a metamorphic protein because it has an intramolecular disulfide bond between Cys11 and Cys48 that restricts conformational freedom. In effect, this disulphide linkage creates a circular polypeptide spanning residues 11–47, against the backdrop of which fold-switching happens. The mechanism by which Ltn fold-switching occurs is still an area of active research. The extensive remodelling of hydrogen bonds and hydrophobic interactions that happens during fold-switching suggests that a disulfide-bonded ‘unfolded state’ must be an intermediate between the two states. Stopped-flow fluorescence-detected kinetics measurements of the temperature dependence of unfolding and interconversion reveal very similar values for the enthalpy and entropy of activation for the two processes, confirming the notion that interconversion must proceed through an ‘unfolded state’ [28]. The authors also measured unfolding rate constants between 0.5 and 5 s^−1^ for Ltn 40 and Ltn10. However, why the unfolding rate constants are so fast when *k*_u_ values for other proteins of similar size are generally one–two orders of magnitude smaller [29], or even what the on-pathway disulfide-bonded ‘unfolded state’ structurally looks like, remains unclear. In contrast to experiments, native-centric Go models [30] as well as replica-exchange simulations [31] have suggested that unfolding may not be necessary for fold-switching, and that interconversion between Ltn10 and Ltn40 can occur via partially unfolded intermediates that differ in the hydrogen bonding patterns of the β-sheet.

The Ltn10 and Ltn40 states of lymphotactin have distinct biological functions within its role as a chemokine, making Ltn a truly metamorphic protein [16,32]. Chemokines are secreted signalling molecules that regulate the migration of leucocytes as part of the immune response to inflammation. In achieving this objective, chemokines bind not only to G protein-coupled receptors (GPCRs) on the surface of leucocytes and stimulate migration, but also to glycosaminoglycans (GAGs) in order to establish a concentration gradient for chemotaxis to occur [24]. However, structure–function correlations are challenging to establish in Ltn because of the co-existence and reversible interconversion of the two structures. Using elegant protein engineering approaches, Volkman and coworkers generated mutants with one extra disulfide bond that are locked in either the Ltn10 (V21C/V59C, CC3-Ltn) [33] or the Ltn40 conformation (A36C/A49C, CC5-Ltn) [32]. While CC3-Ltn is unable to tightly bind GAGs, it is an effective agonist for XCR1; on the other hand, CC5-Ltn binds GAGs with high affinity.

Placed in the context of its family members, the structural and functional characteristics of Ltn appear perplexing. Chemokines routinely dimerize while retaining their chemokine fold [25], but Ltn switches to a novel all-β structure upon homodimer formation. Moreover, chemokines bind to both GAGs and GPCRs using the same chemokine fold, but the Ltn10 form of lymphotactin alone seems to have lost the ability to bind GAGs, and the Ltn40 form is no more a functional agonist of XCR1. Exactly why lymphotactin evolved such a metamorphic behaviour to segregate GAG binding and GPCR activation functionalities into two interconverting conformations remains unclear. However, some clues have emerged from studies on the role of chemokines as defences against viruses, which show that lymphotactin is right at the centre of a fierce and rapidly evolving battlefield between viruses and their hosts. For example, the rat cytomegalovirus genome has developed the capacity to encode for a Ltn-like chemokine that is also able to induce chemotaxis of rat leucocytes [34,35]. Additionally, Ltn is a broad-spectrum inhibitor of HIV-1 and blocks viral entry into cells through a mechanism that requires the novel all-β fold [36,37]. A positively charged cluster in the all-β fold comprising residues Lys42, Arg43, Arg18, Arg35 and Lys46 is necessary for binding the HIV-1 envelope glycoprotein gp120, raising the intriguing possibility that metamorphic features in Ltn may have evolved as defences in the arms race against viral infection.

### 2.2. KaiB

**Figure 3.**
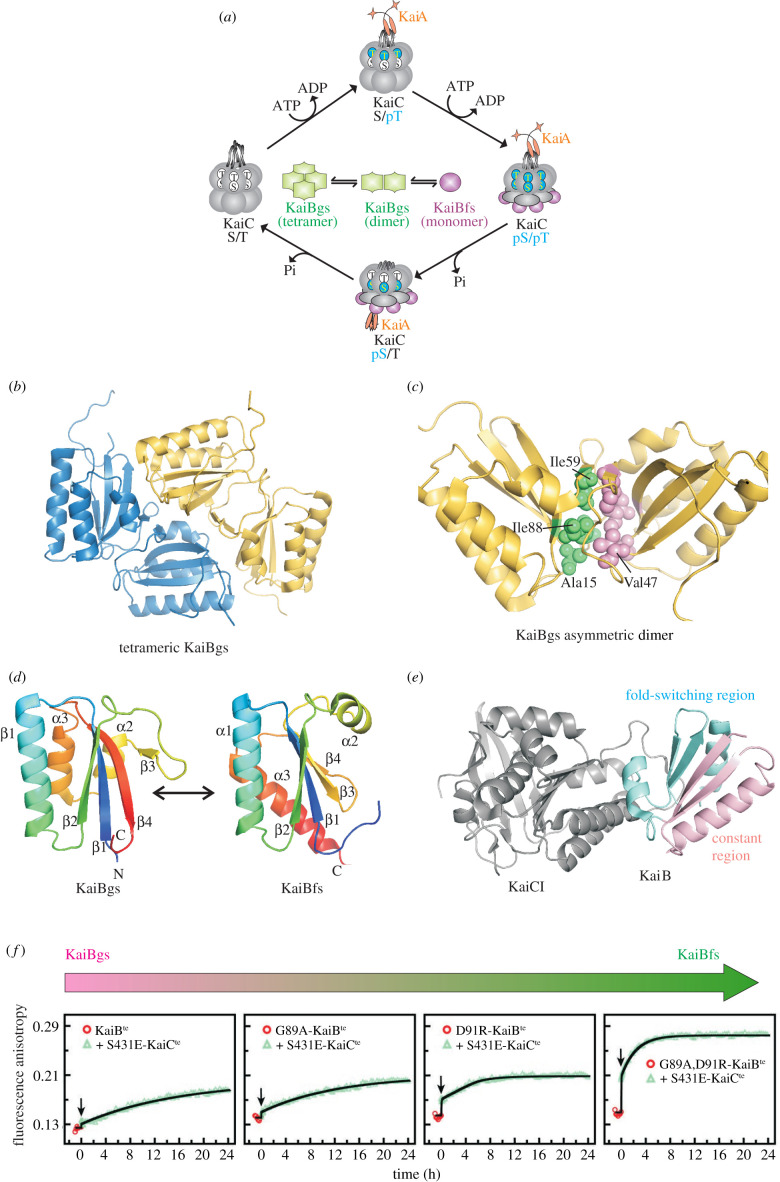
(*a*) The cyanobacterial circadian cycle. KaiC (grey) is a two-domain (CI and CII) protein that organizes into a hexamer. Phosphorylation in the CII domain of KaiC (shown in aqua) at Thr432 and Ser431 helps in establishing the 24 h time period of the cycle. At noon, KaiA (orange) binds to A-loops and stimulates KaiC autophosphorylation. KaiB, which exists as an equilibrium between the tetrameric (green), dimeric (green) and the monomeric fold-switched conformations (purple), binds to the B-loops of the CI domain of KaiC through the thioredoxin-like KaiBfs fold, triggering dephosphorylation in the same order at dusk. After both Thr432 and Ser431 have been dephosphorylated sequentially, the cycle repeats. (*b*) In the ground state, free KaiB is a tetramer of asymmetric dimers (coloured in yellow and blue, PDB ID: 2QKE). (*c*) The asymmetric dimer interface is stabilized primarily by hydrophobic residues such as Ala15, Val47, Ile59 and Ile88 (shown as spheres and coloured green and pink). (*d*) Fold-switching of KaiB results in the conversion of KaiBgs (monomer shown on the left, coloured rainbow) to KaiBfs (PDB ID: 5JYT, right, coloured the same as KaiBgs), which adopts a thioredoxin fold. While the N-terminal segment of KaiB still retains the βαβ secondary structure in both folds, the C-terminal region rearranges from the βααβ in KaiBgs to αββα in KaiBfs. (*e*) The crystal structure of the functional KaiC (CI domain)–KaiBfs complex (PDB ID: 5JWO). The CI domain of KaiC is coloured grey, while the constant region of KaiB is shown in pink and the fold-switching region in cyan. The KaiC–KaiB interface is composed primarily of the C-terminal region of KaiB that undergoes fold-switching. (*f*) Time-course of binding of fluorescently labelled KaiB and its variants with KaiC, followed by fluorescence anisotropy. *Thermosynechococcus elongatus* (te) proteins were used in this study. S431E is a phosphomimetic mutation in KaiC that facilitates binding to KaiB. The binding of wt KaiBgs is monophasic and occurs over the timescale of 24 h. As the fraction of KaiBfs at equilibrium is increased through mutations, the binding becomes biphasic. The fast phase corresponds to the rapid binding of pre-existing KaiBfs to KaiC, while the slow phase corresponds to the rearrangement of KaiBgs to KaiBfs, followed by binding. These experiments indicate that the fold-switching of KaiB is rate-determining in binding to KaiC. Panel (*f*) is reproduced with permission from Chang *et al*. [18].

The cyanobacterial KaiB protein is the only metamorphic protein that has thus far been identified in circadian rhythms. The circadian oscillator in cyanobacteria is composed of three proteins, KaiA, KaiB and KaiC, which generate 24 h rhythms in KaiC phosphorylation through their time-dependent interactions with each other (figure 3*a*) [38–40]. KaiC is a double-ring hexamer, made up of two homologous AAA+ ATPase domains, CI and CII [41]. At the beginning of the day, Thr432 and Ser431 of the CII domain of KaiC are unmodified. Around noon, Thr432 gets phosphorylated (S/pT); in this state, KaiA binds to KaiC through A-loops present at the C-terminus of KaiC to stimulate autophosphorylation [42]. Once Ser431 is also phosphorylated (pS/pT), the CI and CII rings stack onto each other to expose a binding site for KaiB (called the B-loop) on the KaiC CI domain [43–45]. KaiB then binds not only to KaiC but also to KaiA to form a ternary complex (figure 3*a*), sequestering KaiA from KaiC and initiating the dephosphorylation arm of the circadian oscillation [46–48].

In the free form (referred to as the ground state, gs), KaiBgs exists as a tetramer made up of two asymmetric dimers (figure 3*b*, dimer of dimers) [49]. Each of the monomers in the tetramer adopts a novel fold comprising four β-strands and three α-helices arranged in the order βαββααβ. β1 and β2 run parallel to each other, while β4 is hydrogen-bonded and anti-parallel to β1. β3 is a short strand that forms a part of the dimer interface along with the loop connecting β2 and β3, the N-terminal segment of β4 and the C-terminal segment of β1. The interface is predominantly hydrophobic in nature comprising residues such as Ala15, Val47, Ile59 and Ile88 (figure 3*c*); deleting residues 95–108 and simultaneously mutating Tyr8 to Ala and Tyr94 to Ala in tetrameric KaiB generates a dimer where the monomers have the same KaiBgs fold. In its metamorphic counterpart, KaiB exists as a monomer organized into the thioredoxin fold with a βαβαββα secondary structural arrangement (figure 3*d*) [18]. β1, α1 and β2 in the N-terminal half of KaiB do not change significantly in conformation from the ground state (pairwise root-mean-square deviation (RMSD) of Cα between the two structures of 1.3 Å), while the C-terminal half undergoes considerable remodelling during the fold-switching event. Interestingly, most of the interfacial residues of the asymmetric dimer lie in the C-terminal region of KaiB, which undergoes the largest conformational changes upon fold-switching, suggesting that the asymmetric KaiBgs tetramer must fall apart before fold-switching can occur.

The crystal structure of KaiC (CI domain) in complex with KaiB (figure 3*e*) [50] reveals that the interface between KaiBfs and KaiC is made up of several key residues such as Ala15, Val47, Ile59 and Leu60 that become sequestered in the asymmetric dimer interface in KaiBgs. These residues become exposed in the thioredoxin-like KaiBfs and are, therefore, available to bind KaiC, again providing the impression that the protein has been turned inside out. The KaiB metamorphic system is another incisive example of how solvent accessibility of functional residues is modulated by taking advantage of the frustration encoded in conformational free energy landscapes in order to alter the function in different conformations of the same protein.

Very little is known about the mechanism of interconversion between KaiBfs and KaiBgs. Conformational rearrangement between the two states occurs extremely slowly and the extended waiting time required for KaiBgs to convert to KaiBfs contributes to the time delay necessary for maintaining the 24 h circadian period [18]. The kinetics of fold-switching has been estimated only indirectly by probing the binding of KaiB and its variants to KaiC. It is known that only KaiBfs binds KaiC. Fluorescently labelled wild type KaiB exists primarily as KaiBgs (K_eq_(gs–fs) = 0.08) and its binding to KaiC can be described by a single kinetic phase with a time constant of approximately 12 h [18]. In contrast, G89A/D91R KaiB exists predominantly in as KaiBfs (K_eq_(gs–fs) = 6.7) and shows a burst phase as well as a slow phase when binding KaiC. The fast phase is interpreted as the association of KaiC with binding-competent KaiBfs molecules present at equilibrium, while the slow phase corresponds to the interconversion of pre-existing KaiBgs to KaiBfs and its subsequent binding to KaiB. Since the burst phase is significantly faster than the slow phase, approximating the rate-determining step to be the KaiBgs–KaiBfs switching provides an estimate of the interconversion rate constant of the order of 12 h. Three conserved Xaa-Pro linkages, Thr62–Pro63, Leu69–Pro70 and Pro71–Pro72 switch from a trans conformation in KaiBgs to cis in KaiBfs, and this obligate isomerization could be one of the reasons why fold-switching is so slow in KaiB.

### 2.3. IscU

*Escherichia coli* IscU presents yet another variation to the theme of metamorphic proteins, where one of the conformers is structured and the other is partially disordered, but both carry out distinct functions. IscU is a member of the ATP-dependent iron–sulphur cluster (Isc) biogenesis pathway that operates in bacteria and in mitochondria [17]. IscU is the scaffold protein on which Fe–S clusters are assembled before transfer through a specialized Hsp70/Hsp40 system to receiver apoproteins.

**Figure 4.**
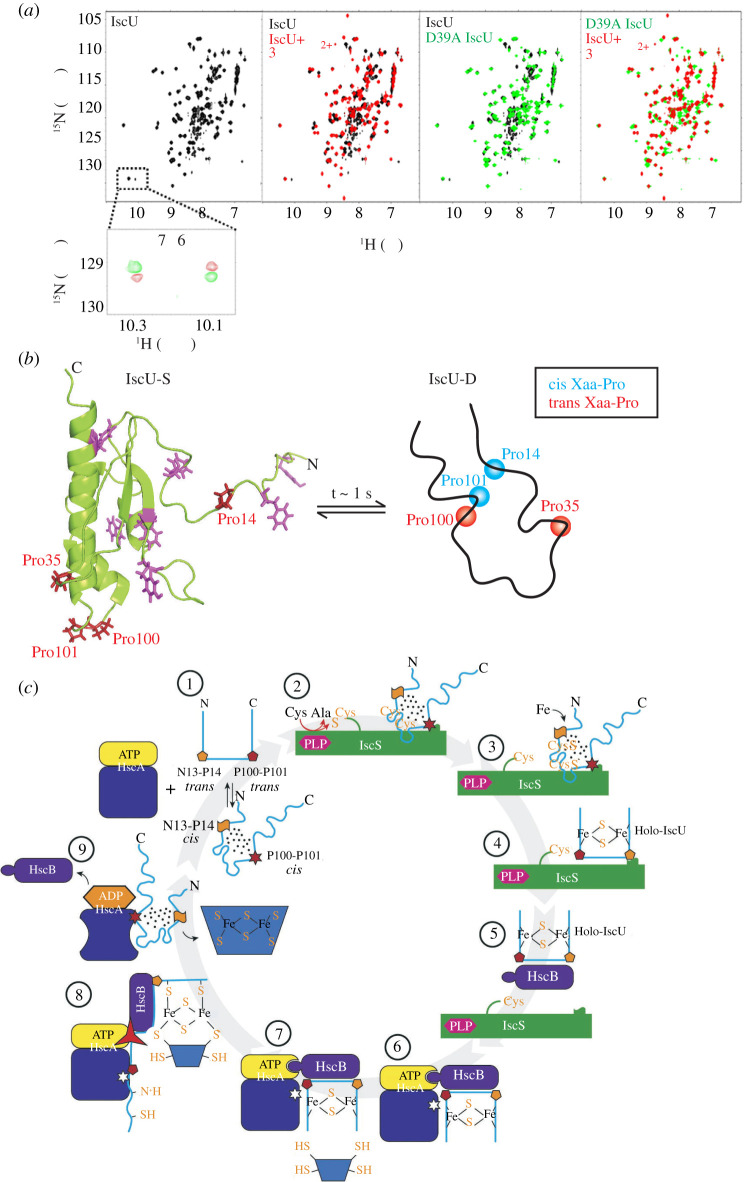
(*a*) (Top) ^1^H–^15^N HSQC spectra of IscU (left) showing duplicated peaks for the lone Trp76 sidechain (dotted box) that indicate the co-existence of two conformations in the sample. Overlays of spectra (middle) of IscU in the presence of 3 mM Zn^2+^ (red) or D39A IscU (green) with apo wt IscU (black) show that the minor Trp sidechain resonance disappears in both cases. This indicates that Zn^2+^ and the D39A mutation stabilize the same conformation (IscU-S). (Bottom) ^15^N magnetization exchange spectrum of apo wt IscU that shows cross-peaks (red) between the diagonal sidechain resonances originating from Trp76 in the two co-existing conformations of IscU. The appearance of these cross-peaks demonstrates that IscU-S and IscU-D interconvert reversibly on the ms–s timescale. (*b*) Cartoon representation of the NMR structure of IscU-S (PDB ID: 2L4X). The four Pro residues in IscU-S are shown as red sticks, while the aromatic residues are coloured magenta. Curiously, most of the aromatic residues are solvent-exposed and do not contribute to the hydrophobic core of IscU-S. The structured IscU-S is in equilibrium on the ms–s timescale with a partially disordered form, IscU-D. Two of the Xaa-Pro linkages in IscU-D, Asn13–Pro14 and Pro100–Pro101 are in cis conformation, while all four Xaa-Pro bonds in IscU-S are in the trans form. (*c*) The role of the scaffold protein IscU in the functional Fe–S cluster biogenesis cycle. (1) IscU exists in two conformations, IscU-S and IscU-D. (2) The pyridoxal phosphate (PLP)-dependent enzyme IscS catalyzes the conversion of Cys to Ala and in-turn generates persulfides on IscU-D by preferentially binding to it. Once Fe is introduced into this complex (3), fold-switching occurs and the IscU-state is stabilized by the Fe–S cluster. The J-domain co-chaperone, HscB, recognizes IscU-S and facilitates the assembly and transfer of the Fe–S cluster to the downstream acceptor protein (5–9). During this process, IscU is transferred to the Hsp70-like HscA, which stabilizes the partially disordered IscU-D form of IscU. Upon release from HscA, free IscU then equilibrates again into ordered and partially unfolded forms to reset the cycle. Panels (*a*) and (*c*) are reproduced with permission from Markley *et al*. [17].

The existence of two IscU conformations in thermal equilibrium can be seen clearly from the ^1^H–^15^N heteronuclear single quantum correlation (HSQC) spectrum of IscU (figure 4*a*, left), which displays two sets of resonances in slow exchange on the NMR chemical shift timescale for several residues, including the sidechain NH of the lone Trp in IscU [51]. ^15^N magnetization exchange experiments unequivocally demonstrate that the two forms interconvert reversibly with rate constants *k*_SD_ = 0.8 s^−1^ and *k*_DS_ = 2.0 s^−1^ at 25°C (figure 4*a*, bottom) [52]. The stabilities of the structured and disordered forms are approximately equal at 37°C (*p*_S_ = 0.4, *p*_D_ = 0.6, 150 mM NaCl, pH 8), while IscU-S is maximally stable at 25°C [53]. The structured form of IscU (IscU-S) is made up of a three-stranded β-sheet at the N-terminus and a four-helix-bundle downstream that docks onto the β-sheet (figure 4*b*) [54]. Three of the four helices are short (two turns), while the C-terminal helix is long with seven turns. The hydrophobic core of the protein is formed primarily from Ile, Leu and Val residues with a few interdigitating Ala. Interestingly, the six aromatic amino acids in IscU are significantly solvent-exposed and none of them participate in the core (figure 4*b*, left) of IscU-S. While the NMR spectrum of IscU-S is well-dispersed, a large fraction of the peaks belonging to IscU-D resonate at the random coil chemical shift, indicating that IscU-D has both disordered and ordered regions (figure 4*a*, left). This observation is supported by ^1^H–^15^N heteronuclear nuclear Overhauser enhancement (NOE) values, which are negative for the residues falling in the random coil region and positive otherwise [53].

Apart from the order-partial disorder transition, the most prominent structural change that occurs during fold-switching of IscU is the change in the stereochemistry of the Xaa-Pro bonds of two conserved Pro residues Pro14 and Pro101 from trans (IscU-S) to cis (IscU-D) (figure 4*b*) [53]. Given that Pro14 is in the disordered N-terminus of IscU-S, we do not yet understand the nature of interactions that stabilize it in the cis form in IscU-D. It is possible that the cis Asn13–Pro14 bond brings the N-terminus closer to the rest of the protein and buries hydrophobic surface area to stabilize the cis Pro14 conformation. Unlike in KaiB, where Pro cis–trans isomerization of three Xaa-Pro bonds likely slows down fold-switching to the timescale of hours, the isomerization of two such bonds in IscU occurs within a second. Considering that the rate constant of a single Pro cis–trans isomerization typically occurs 1–2 orders of magnitude slower in model peptides [55] than in IscU, the reason for this speed-up in IscU remains unclear.

The two metamorphic states of IscU have distinct functions just as in the case of lymphotactin (figure 4*c*). IscU-D selectively binds to the cysteine desulfurase [52], IscS, which is a pyridoxal phosphate-dependent enzyme that generates and transfers a sulfur to Cys residues in IscU to form persulfides. Subsequent formation of the Fe–S cluster induces fold-switching by stabilizing the ordered form of IscU, which migrates from IscS to HscB (DnaJ-like Hsp40) in the same conformation. IscU remains in the structured state until the Fe–S cluster is relayed to the acceptor protein and IscU-S is transferred back to HscA/ATP via HscB. The structured scaffold is necessary to stabilize the nascent Fe–S cluster before transfer to the apoprotein [17]. On the other hand, since HscA (DnaK-like Hsp70) binds disordered and partially ordered species, IscU switches folds from IscU-S to IscU-D and is released back into the Fe–S biogenesis pathway in the disordered conformation. Thus, while IscU-S binds IscS and HscB by virtue of its structure, IscU interacts with HscA in the partially ordered IscU-D form due to the constraints imposed by the binding pocket of HscA, providing a functional context for the metamorphic behaviour.

### 2.4. Mad2

The metamorphosis of Mad2 plays an integral role in establishing the protein–protein interactions that form a part of the cell-cycle surveillance mechanism called the spindle checkpoint complex [56,57]. During mitotic cell division, the alignment of chromosomes at the metaphase plate occurs via the attachment of the kinetochore in each sister chromatid to microtubules originating from opposing spindle poles. Incomplete attachment of sister chromatids to the mitotic spindles during metaphase can result in aneuploidy in the daughter cells and this is prevented by the spindle assembly checkpoint (SAC). The transition from metaphase to anaphase is mediated by the anaphase-promoting-complex APC/C, which is a ubiquitin ligase that polyubiquitinates securin and cyclin B. Subsequent degradation of securin activates separase, which cleaves the cohesin complex that holds the sister chromatids together. In turn, unattached kinetochores at the metaphase plate activate SAC, which inhibits APC/C and delays the onset of anaphase.

**Figure 5.**
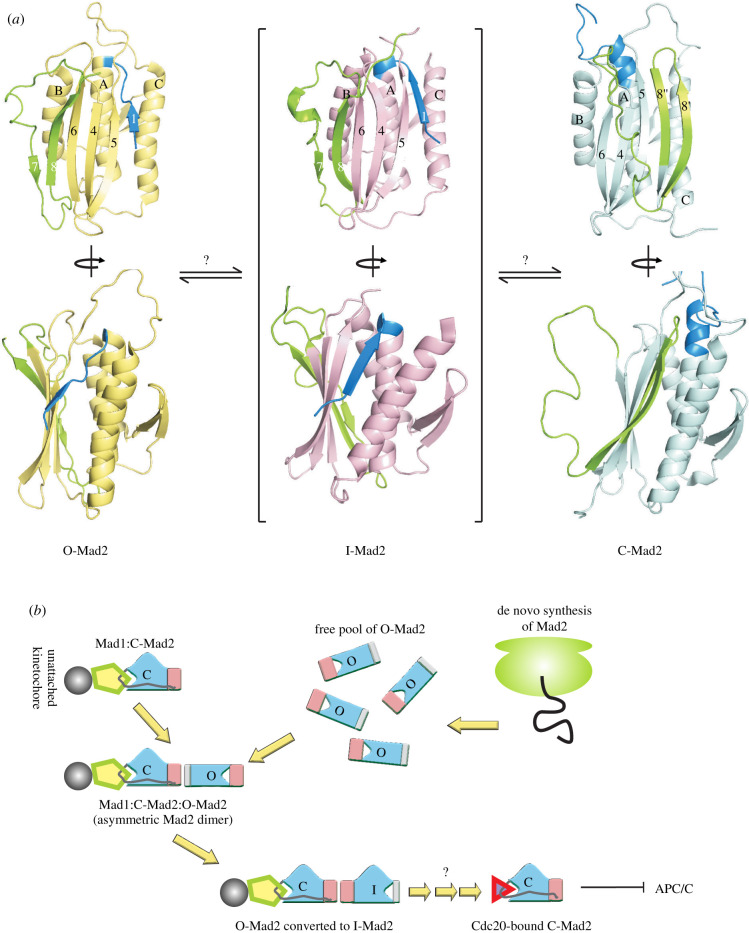
(*a*) Orthogonal views (top and bottom) of the cartoon representation of O-Mad2 (left, PDB ID: 1DUJ), I-Mad2 (middle, PDB ID: 3GMH) and C-Mad2 (right, PDB ID: 1S2H). The N-terminal and C-terminal regions undergoing fold-switching are shown in blue and green, respectively, while the core that remains unchanged is indicated in pale yellow. Strands and helices are denoted with numerals and upper case letters, respectively. The bottom view shows that the helix orientation of I-Mad2 resembles that of C-Mad2, though the overall fold is similar to that of O-Mad2. (*b*) The functional cycle of Mad2 within the spindle checkpoint complex. Free de novo synthesized Mad2 exists primarily as O-Mad2, while the C-Mad2 in cells exists as a tight complex with Mad1. The Mad1–C-Mad2 complex is recruited to unattached kinetochores and the C-Mad2 forms an asymmetric homodimer with O-Mad2. This C-Mad2–O-Mad2 interaction converts O-Mad2 to I-Mad2 and subsequently activates it to recognize the cognate binding partner, Cdc20. Sequestration of Cdc20 inhibits the APC/C ubiquitin ligase and prevents the premature separation of sister chromatids.

Arguably the first metamorphic protein to be discovered, the 25 kDa Mad2, is a central component of the SAC that folds into a HORMA domain (named after the **Ho**p1p, **R**ev7 and **Ma**d2 proteins) [58]. The core structure of Mad2 has three α-helices and a β-hairpin between helices A and B, as well as a three-stranded anti-parallel β-sheet (figure 5*a*). This core is preserved during the metamorphic transition between C-Mad2 [59] and O-Mad2 [60], during which a second β-hairpin moves from one side of the β-sheet to the other. This C-terminal β-hairpin, comprising strands 7 and 8, hydrogen bonds with strand 6 in O-Mad2 (figure 5*a*, left), while the N-terminus of the protein forms a short β-strand that aligns with strand 5. Upon conformational interconversion, the C-terminal hairpin (labelled as strands 8′ and 8″) displaces the N-terminal β-strand to hydrogen bond with strand 5, while the N-terminal β-strand rearranges into two extra turns of helix A (figure 5*a*, right). There is thus a dramatic alteration in the hydrogen bond network in the periphery of the protein, similar to what is observed in Ltn.

While much of the structural basis underlying folding and interconversion in the Mad2 metamorphosis remains to be elucidated, glimpses of the complexity in the folding process have begun to emerge. Similarly to KaiB, the O-Mad2–C-Mad2 rearrangement occurs extremely slowly, on the timescale of several hours, with the forward reaction taking 9 h, and the backward reaction 6 times longer [59]! C-Mad2 is approximately 10-fold more stable than O-Mad2 [59], though the exact number varies depending on the source and has been difficult to establish partly because of the irreversibility in thermal denaturation experiments, and partly because of the long times necessary for the O-Mad2–C-Mad2 arm to reach equilibrium [61]. In sharp contrast to the slow interconversion kinetics, fluorescence-detected stopped-flow measurements have shown that the folding and unfolding of O-Mad2 and C-Mad2 occur on a timescale of seconds (*k*_f_(C-Mad2) = 11 s^−1^, *k*_f_(O-Mad2) = 6 s^−1^, *k*_u_(C-Mad2) = 0.046 s^−1^, *k*_u_(O-Mad2) = 0.086 s^−1^) [61]. These experiments lend support to the idea that interconversion between O- and C-Mad2 occurs via local or global unfolding to a high energy conformation, followed by kinetic partitioning and refolding to either the O- or the C-Mad2 forms [62].

Mad2 exemplifies the sophistication that can be observed in the protein folding kinetics, thermodynamics and pathways, and how cells can put these complexities to use. For instance, one of the most fascinating questions about metamorphic proteins is the triage between the alternate protein conformations that accompanies de novo protein folding. Virtually all of the free Mad2 inside cells is present as kinetically trapped inactive O-Mad2 generated as a result of de novo protein folding and unable to convert to C-Mad2 because of the slow interconversion between the two forms [19,59]. Therefore, the C-Mad2/O-Mad2 system inside cells appears to exist in a non-equilibrium state (figure 5*b*). By contrast, cellular C-Mad2 is found tightly bound to Mad1 at kinetochores upon checkpoint activation and fluorescence recovery after photobleaching (FRAP) data have demonstrated that there is little or no turnover of this complex [63–66]. Given that the downstream target of Mad2, Cdc20, binds at the same location as the upstream activator, Mad1 [67,68], models describing the recognition of Cdc20 by Mad2 face a conundrum: on the one hand, the Cdc20-binding-competent conformation resembles C-Mad2, and all C-Mad2 molecules are sequestered by Mad1; on the other hand, all of the free Mad2 is trapped in the inactive O-Mad2 conformation that does not convert to C-Mad2 on the timescale of checkpoint activation. Elegant work from several labs has demonstrated that this conundrum is resolved by the ability of the Mad1–C-Mad2 complex to catalyze the conversion of latent O-Mad2 to C-Mad2 and thereby activate Mad2 to bind Cdc20 [60,66,68–73]. Recently, the structural features of an intermediate C-Mad2–I-Mad2 complex have been solved using a combination of X-ray crystallography and NMR spectroscopy [74] that reveal how I-Mad2 retains the O-Mad2 fold but has a core and helix-bundle orientation that resembles C-Mad2 (figure 5*a*, middle), raising the intriguing possibility that I-Mad2 may be an on-pathway intermediate connecting O- and C-Mad2.

Post-translational modifications within the cellular milieu offer another way to modulate conformational equilibria in metamorphic proteins, as seen in the case of Mad2. Phosphorylation of Mad2 at Ser195, as well as the phosphomimetic mutation S195D, inhibits the conformational transition of O-Mad2 to C-Mad2 as observed from time-dependent 1D NMR spectra of the wt protein and its variants [75]. The functional consequence of this inhibition is that S195D Mad2 does not bind its cognate ligand Cdc20, and the expression of this variant inside cells results in deficiencies in spindle checkpoint regulation.

### 2.5. RfaH

**Figure 6.**
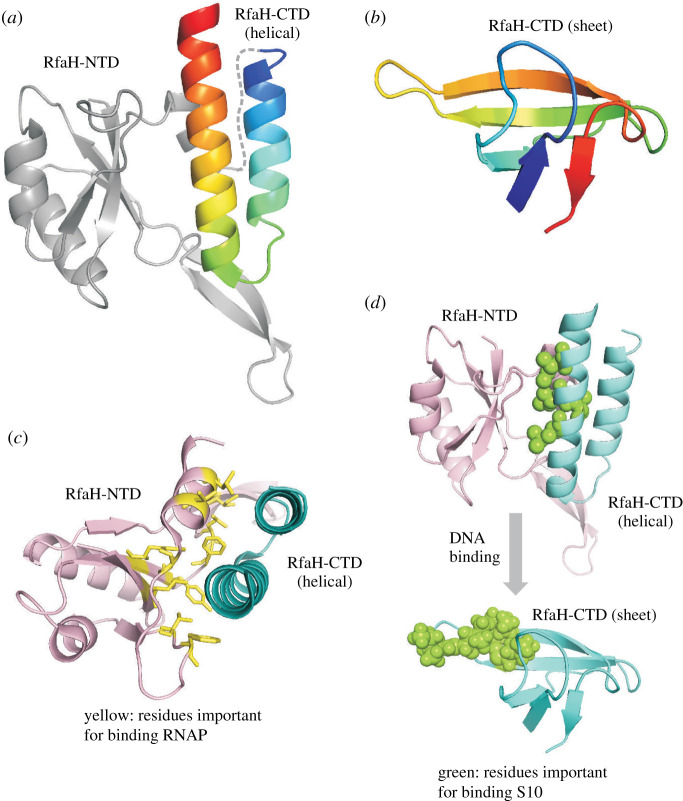
(*a*) Cartoon representation of the X-ray structure of full-length wt RfaH (PDB ID: 2OUG). The N-terminal domain (NTD) is shown in grey, while the fold-switching C-terminal domain (CTD) is coloured rainbow. In free full-length RfaH, the CTD is helical. (*b*) The all-β sheet structure of the CTD of RfaH obtained using NMR spectroscopy (PDB ID: 2LCL). The colour scheme is identical to the CTD in (*a*). (*c*) The residues in the NTD (pale pink) important for binding RNA polymerase are shown as sticks on the structure of RfaH and coloured yellow. Most of these residues are sequestered by the helical CTD (cyan) in the free form of RfaH, but become exposed upon fold-switching and detachment of the CTD from the NTD. (*d*) The residues in the CTD (cyan) important for binding the ribosomal protein S10 are shown as green spheres in both the helical (top) and sheet conformations of the CTD. Most of these residues are also buried at the NTD–CTD interface but become available for binding S10 when the NTD interacts with RNA polymerase.

The *E. coli* virulence factor RfaH is a textbook example of how interdomain interactions can modulate protein conformation (figure 6*a*,*b*). RfaH is a paralog of NusG and both proteins have structurally similar N-terminal domains (NTDs) that bind to RNA polymerase (RNAP) and switch it into a pause-resistant processive mode [76,77]. The C-terminal domain (CTD) of NusG is a β-barrel that remains independent from the NTD and interacts with the transcription termination factor Rho [78–80]. In contrast, the CTD of free RfaH (RfaH_C_) folds into an α-helical hairpin (figure 6*a*) that binds to the NTD and sequesters the RNAP recognition motif (figure 6*c*) [81]. When RfaH binds DNA, the NTD and CTD dissociate from each other, enabling the NTD to associate with RNAP [81,82]. Simultaneously, the CTD spontaneously rearranges from an α-helical hairpin into a five-stranded β-barrel (figure 6*b*) that closely resembles the CTD of NusG [83]. The remodelled CTD of RfaH then interacts with the ribosome to activate translation (figure 6*d*), generating a bridge that couples transcription and translation. The fold-switching that occurs when RfaH_C_ dissociates from the NTD demonstrates the importance of interdomain interactions in stabilizing the α-helical fold of the CTD [82].

The RfaH system offers yet another variation to the theme of metamorphic proteins as it involves a multidomain protein where one of the domains acts as a scaffold for the metamorphosis of the other domain [83]. Similar to Ltn, there is a global conformational change in RfaH_C_ as it switches from the autoinhibitory α-helical conformation to the functionally active β-sheet form. The β-sheet conformation contains five β-strands arranged in the order β5(F158–K160)–β1(K115–I118)–β2(Q127–F130)–β3(R138–N144)–β4(E149–K155). RfaH_C_ by itself exists in the β-sheet conformation. In order to demonstrate that fold-switching also happens in the context of full-length RfaH, Rösch and colleagues created a variant E47S, in which a key electrostatic linkage between Glu47 and Arg138 tethering the NTD and CTD is disrupted [83]. Peaks arising from both the α-helical and β-sheet conformations with an intensity ratio of approximately 1 : 1 can be seen in the ^1^H–^15^N HSQC spectrum of E47S RfaH, showing that the helical and sheet structures can coexist.

While experimental studies probing the interconversion of the helical and sheet forms of RfaH are lacking, there are a number of computational studies addressing this question using methods such as targeted molecular dynamics [84], Markov state modelling [85], replica exchange [86–88] and quasi-continuous interpolation [89], coarse-grained simulations [90], dual-basin structure-based models [91] and pulling simulations [92]. There is general agreement that interconversion is triggered by the fraying of the N-terminal α-helix. Some studies have suggested that subsequent remodelling occurs through a high energy ensemble of disordered conformations [85–87,89] that form the five-stranded β-sheet in due course through a series of states with one or more β-hairpins. However, the coarse-grained structure-based model predicts that there are two on-pathway intermediates facilitating the fold-switching, with one of them resembling the helical and the other, the sheet conformations [91].

## 3. Evolutionary importance of metamorphic protein systems

The study of metamorphic proteins is relevant not only because these proteins leverage complex conformational free energy landscapes for function, but also because of the insights they promise to offer into protein evolution. Viewed from this evolutionary eyepiece, metamorphic proteins are significant in three ways. First, metamorphic proteins provide evidence at the molecular level for the escape from adaptive conflict (EAC) [93] model of protein evolution [94–96]. Adaptive conflict is a fundamental conundrum in molecular evolution that describes the difficulty faced by a protein in adapting to a new function while still retaining its ancestral function. In models such as neofunctionalization, adaptation occurs after a gene duplication event, with one gene copy retaining the ancestral function and the second copy evolving to perform the new function [97]. On the other hand, EAC is a model where multifunctional (or moonlighting) proteins facilitate adaptive processes both before and after gene duplication [98]. With the discovery of metamorphic proteins, we now know that proteins can adopt structurally distinct yet reversibly interconverting folds that directly result in multifunctionality. Such conformational heterogeneity can, therefore, enable protein evolution by expanding the functional repertoire of a limited set of protein sequences [99–103].

Second, metamorphic proteins are bridges between two distinct protein folds and represent intermediate states in mutational paths connecting the two structural topologies. In this context, they are uniquely placed as model systems for computational [104] and experimental [105] studies of mechanisms underlying evolutionary dynamics, the role of selection pressure and the impact of mutations.

Finally, how metamorphic proteins evolved and what their ancestors looked like is, by itself, a fascinating question. In an elegant recent study [106], Volkman and coworkers have addressed some of these questions by using phylogenetic ancestral reconstruction methods to generate sequences of ancestors of Ltn and characterizing them using NMR spectroscopy. Ltn evolved from an ancestor that had the chemokine fold. Loss of the second conserved disulfide linkage present in chemokines was one of the earliest events in its evolution, though this was not sufficient for the emergence of metamorphosis. Subsequent ancestors were metamorphic, but the symmetric population of the two folds was one of the final events to occur in Ltn evolution. One of the most striking conclusions about this report is that Ltn does not appear to be an evolutionary intermediate transitioning from the chemokine fold to the novel dimeric Ltn40 fold, but rather seems to have evolved to remain metamorphic. This suggests that metamorphic proteins do not necessarily have to be missing links in protein evolution, but can represent end points in evolution as well.

## 4. Sequence-based prediction of metamorphic proteins

The challenges inherent in systematically identifying metamorphic proteins using experimental approaches, as opposed to their serendipitous discovery, have raised the intriguing question of whether metamorphosis can be predicted based on protein sequence alone. While this question is difficult to address because very few truly metamorphic protein sequences are available, two distinct approaches have been adopted to develop such computational algorithms.

The first approach uses a biophysical hydrophobic-polar model to coarse-grain the protein sequence [104]. Protein sequences are treated as strings of 18 beads that can be hydrophobic or polar. The conformation of this string is described as a self-avoiding walk on a two-dimensional lattice grid. Each intra-chain contact between pairs of hydrophobic beads is assigned a favourable energy that serves to drive the folding of this string. The native state degeneracy of a sequence is then defined as the number of native states that have the same number of hydrophobic contacts. Within this definition, sequences with a degeneracy value larger than 1 are classified as metamorphic (or bi-stable). Using this definition, Chan and coworkers [104] have identified 181 bi-stable proteins in the protein conformational database [107], which is a collection of proteins from the PDB for which more than one structure has been reported. An interesting candidate among this predicted set is spinach chloroplast thioredoxin (PDB ID: 1FB6 [108], 1GL8 [109]), where the N-terminal segment of the second α-helix in 1FB6 is bent into two 3_10_ helices in 1GL8 that are oriented at approximately 110° and 90° to the C-terminal half of the helix. The thioredoxin fold is already known to be involved in the metamorphosis of KaiB [18], though the first helix in KaiB remains unchanged in conformation in the two folds of KaiB. In contrast, this helix is structured differently in the predicted metamorphs of spinach thioredoxin, suggesting that the ancient thioredoxin fold may have undergone more than one metamorphic transition during evolution.

The second class of approaches is based on the premise that metamorphic proteins should confuse secondary structure prediction algorithms [110–112]. The output of sequence-based secondary structure prediction algorithms is expected to be ambiguous for regions of metamorphic proteins that switch conformation in the two folds, and this ambiguity has been leveraged to predict protein metamorphosis. Porter & Looger [110] have used inconsistent secondary structure predictions, in conjunction with the existence of independent folding units within proteins (identified using the structure energy equivalence of domains (SEED) algorithm) to detect approximately 100 fold-switching proteins registered in the PDB, as well as another approximately 100 proteins which have only one solved structure, but which nevertheless are predicted to switch folds. Similarly, Wang and coworkers [112] have constructed a model trained on 201 metamorphic and 136 monomorphic protein structures from the PDB, and this model classifies proteins as monomorphic or metamorphic based on the sequence. It must be noted that both of these studies define all fold-switching proteins as metamorphic, while the metamorphic proteins described in this article exist in multiple folded states in the absence of ligands or cofactors. While the false positive rate for sequence-based prediction of metamorphic proteins remains high (10–30%), these algorithms represent important first steps in addressing this challenging problem. The interesting candidates that emerge from this second approach are a number of viral proteins such as bacteriophage T7 and SARS glycoproteins, as well as pore-forming toxins that insert into membranes, though there is as yet no experimental validation of these predictions.

## 5. Methods for studying metamorphic proteins

The co-existence of multiple conformations in equilibrium has posed a significant challenge in detecting and characterizing metamorphic proteins. Crystallographic and NMR structure determination pipelines are generally optimized to weed out such heterogeneous systems, and this implicit bias in sample preparation may be the cause for why the list of known metamorphic proteins remains so short. Ever since the existence of proteins with multiple folded conformations was established, however, a number of experimental and computational tools have come together to foster our understanding of metamorphic protein systems. The main questions that have been addressed include the structures of the two conformations, the mechanism of interconversion, the investigation of the functions of the different conformers, and some tantalizing glimpses into the evolution of metamorphic proteins.

Both X-ray crystallography and NMR spectroscopy have proven to be equally important in elucidating the structures of metamorphic proteins locked in a particular conformation. While the structures of Ltn10 [26], Ltn40 [16], KaiBfs [18], O-Mad2 [60], C-Mad2 [59], IscU-S [51], IscU-D [52] and RfaH (all-β) [83] were solved using NMR spectroscopy, the structures of KaiBgs [49] and RfaH (all-α) [81] were determined using X-ray crystallography. The structural features in these systems have also been identified and validated using complementary methods such as mass spectrometry [113–115]. Multidimensional NMR spectroscopy has been at the forefront of research in this area because of its ability to provide atomic resolution information on systems that exhibit static and dynamic heterogeneity [116–118]. NMR has been pivotal in establishing the presence of multiple conformations in all of the metamorphic proteins described in this review, as well as in unequivocally demonstrating the reversible interconversion of these conformations in Ltn and IscU. Moreover, we anticipate that the newly developed NMR methodology for structurally characterizing sparsely and transiently populated biomolecular conformations [10], such as chemical exchange saturation transfer [119,120] and relaxation dispersion [121,122], will help further our understanding of the mechanisms underlying conformational interconversion in these systems.

Mechanistic studies probing the atomic details of fold-switching have thus far remained primarily within the domain of computational methods. The small (approx. 100 residue) size of many of these proteins makes them amenable to sophisticated molecular dynamics methods without placing a heavy burden on computational resources. Coarse-grained, as well as all-atom molecular dynamics (MD) simulations have been helpful in mapping the free energy landscape and kinetic pathways of Ltn [114], Mad2 [123] and RfaH (see above). The conflicting predictions from computational methods in the case of RfaH, however, have made it clear that such mechanistic studies will benefit from experimental measurements. Surprisingly, single-molecule methods have not been extensively employed to probe metamorphic proteins. On the other hand, ion-mobility mass spectrometry of Ltn has revealed that Ltn10 is considerably more flexible than Ltn40 [114].

A number of innovative protein engineering methods have also been used to dissect the functions of the two folds of metamorphic protein systems. Functional studies are fraught with difficulty when two conformations rapidly and reversibly interconvert, as it becomes tricky to ascribe a protein–protein or protein–ligand interaction to one of the conformers. Volkman and coworkers have constructed locked versions of Ltn10 [33] and Ltn40 [32] by incorporating additional disulfide linkages to stabilize one or the other conformer, thereby generating an artificial kinetic barrier for the interconversion. Structure-guided mutations that selectively stabilize one conformation are also available for all the other metamorphic protein systems discussed here, providing an arsenal of variants for understanding structure–function relationships.

## 6. Conclusion and perspectives

Protein dynamics is central for function and has been implicated in malfunction and disease. The conformational heterogeneity observed in proteins is a consequence of the frustration inherent in protein conformational free energy landscapes. No other system illustrates the malleability of these landscapes better than metamorphic proteins, which have evolved to populate two distinct structural tertiary folds capable of handling different functions.

Our current understanding of metamorphic proteins stems from complementary structural and evolutionary approaches ranging from NMR spectroscopy and X-ray crystallography to molecular dynamics simulations and ancestral reconstruction. We have been able to elucidate the structures and functions of the different conformers and also establish, in some cases, their reversible interconversion. It is clear from the available data that the study of metamorphic proteins is crucial not only to understand structure–function relationships, but also because metamorphic proteins are excellent model systems in areas as diverse as structural and evolutionary biology.

Nevertheless, the field of metamorphic proteins remains in its infancy 12 years after the influential article of Murzin coining the term ‘metamorphic protein’ [12]. Typical metamorphic proteins characterized thus far show only two conformational states in equilibrium. Whether this is a limitation of the methodology we have available at our disposal to detect multiple different conformations, or if it is an inherent constraint posed by the protein-free energy landscape remains unclear, and the degree of structural plasticity that can be exhibited by a metamorphic protein is still an open question. Indeed, there are suggestions in the literature that HIV-1 reverse transcriptase can adopt three different structures [23]. There is an urgent need to develop robust sequence-based predictions of metamorphic protein systems that can then be characterized experimentally. This will pave the way to augment our list of metamorphic proteins and to conduct systematic studies to recognize themes and patterns. We also understand very little about the mechanism of interconversion of the distinct structures and how compact folded conformations interconvert efficiently and reversibly without aggregating. Finally, where do metamorphic proteins fit in the broad fabric of protein evolution? Are they intermediates caught in the act, or are they sophisticated evolutionary endpoints sculpted by selection pressure to favour multiple folded conformations? These are some of the exciting questions faced by the field of metamorphic proteins, the answers to which will further our quest for deeper insights into biomolecular structure, dynamics and evolution.

## Data accessibility

This article has no additional data.
